# Unveiling the inner structure of electron pulses generated from a laser-wakefield accelerator

**DOI:** 10.1038/s41377-023-01269-1

**Published:** 2023-09-12

**Authors:** Malte C. Kaluza

**Affiliations:** 1 Institute of Optics and Quantum Electronics, Max-Wien-Platz 1, 07743 Jena, Germany; 2 Helmholtz-Institute Jena, Fröbelstieg 3, 07743 Jena, Germany

**Keywords:** Nonlinear optics, Plasma-based accelerators

## Abstract

A novel diagnostic method has been used to gain deeper insight into the transverse structure and its evolution of electron pulses generated from a laser-wakefield accelerator.

Over the last century, particle accelerators have helped us to shape our understanding of nature. They are currently used in a large number of cases for fundamental science and for various applications. The most fundamental discoveries and applications in basic science, e.g., the development and the experimental proof of the standard model of particle physics, culminating in the discovery of the Higgs Boson (for which the Nobel Prize in physics was awarded in 2013), or the generation of ultra-short X-ray pulses for resolving structures and dynamics on sub-atomic length- and time-scales have only been possible with state-of-the-art lepton and hadron accelerators. These accelerators have been developed and optimized towards their intended applications and they have been and still are an impressive success story, proven by numerous discoveries over the last century.

However, most of these accelerators are large in size and thus are extremely expensive in installation and daily operation. Therefore, they only can provide limited beam time and therefore limited access to users. Furthermore, many current or future applications either call for advanced particle pulse parameters, e.g., higher kinetic energies, shorter pulse durations, or higher peak or average currents, or they would benefit from more compact accelerator installations, which would potentially allow for less-restricted access to these machines. In any case, these requirements motivate the ongoing quest for possible candidates for novel particle accelerator technologies.

Plasma-based particle accelerators are one of such potential candidates for the next generation of particle accelerators. For the acceleration of electrons in such a plasma-based accelerator, a so-called plasma wave (or the plasma wakefield)^1^, which is a periodic plasma-density structure co-moving with a driving pulse at velocities close to the speed-of-light, can provide the required acceleration fields (up to several GV/m) over a sufficiently long acceleration length (millimeters to several 10’s of centimeters). As the driver for the wakefield, either high-power laser pulses^2–4^ or relativistic electron^5^ or proton pulses^6^ can be used. The generated electron pulses have kinetic energies up to several GeV^7^, pulse durations as short as a few femtoseconds^8,9^, initial source sizes of micrometers only^10,11^, and ultra-low emittances^12^. Recently, such electron pulses generated from plasma-based accelerators have successfully been used to seed free-electron lasers at different labs worldwide^13–15^, proving their huge potential for further applications.

However, the generated electron pulses still suffer from significant shot-to-shot fluctuations, which makes their further application much more challenging. For their stabilization, a better understanding of the acceleration process and a detailed knowledge of the critical experimental parameters is essential. While large-scale numerical simulations can help to describe the interaction and gain insight, in experiments this can only be achieved by applying well-adapted diagnostic tools, which need to be able to resolve space and time structures on the order of micrometers and femtoseconds, respectively, i.e., the characteristic time- and space-dimensions of the electron pulses themselves.

While over the last decade, several high-resolution diagnostic techniques have been developed and successfully applied for the accelerating structure in the plasma (the wakefield)^16–21^, only a few diagnostic tools exist that can detect the electron pulses and measure their parameters and their evolution with sufficient resolution in the plasma itself^9^. In their recent paper “Femtosecond electron microscopy of relativistic electron bunches”, Yang Wan and co-workers from the Weizmann Institute of Science in Rehovot, Israel, have successfully applied a novel diagnostic technique, which is capable of measuring several critical parameters of the electron pulses generated in a laser-wakefield accelerator in the plasma itself, both in a non-invasive manner and in a single shot^22^. This diagnostic can be used to measure the wakefield but—more importantly—also the accelerated electron pulse from a first laser-wakefield accelerator (driven by the pump pulse) and derive several parameters, which have not been accessible so far. For the measurement of these electron pulse parameters, a second relativistic electron pulse, generated from a second laser-wakefield accelerator, which was driven by a second, synchronized laser pulse, is applied (see Fig. 1, top).

**Fig. 1:**
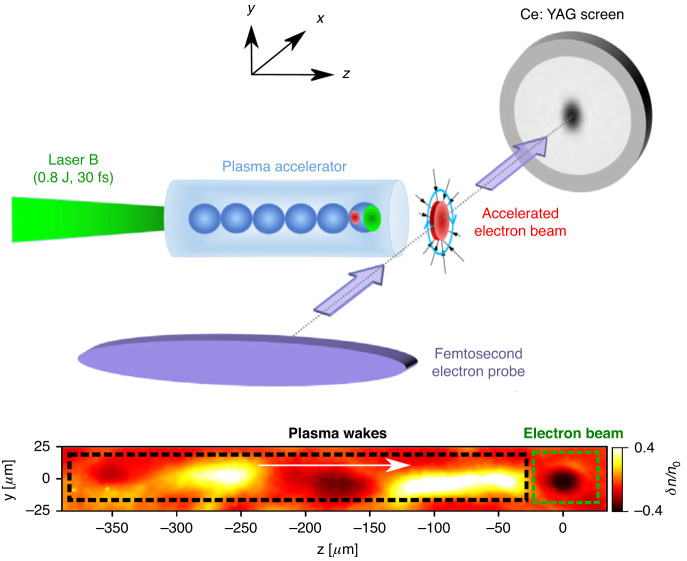
Principle setup and exemplary results. Top: Principle sketch of the experimental setup for the transverse probing of electric and magnetic fields in a laser-plasma accelerator and of the accelerated electron pulses with a second, transverse electron probe pulse. Bottom: Electron probe image of the laser-driven plasma wake (seen on the left in the dashed black region) and of the accelerated electron beam (in the dashed green region on the right). Both images are adapted from ref. ^22^

This second electron pulse (called the probe pulse) propagates transversely through the interaction region of the laser-wakefield accelerator driven by the first (the pump) laser pulse. The acceleration fields generated in this plasma wave but also the electric and magnetic fields generated by the electron pulse itself deflect the electrons in the probe pulse laterally, which—after several centimeters of propagation—leads to intensity variations in the beam profile of the electron probe beam, which can then be detected (see Fig. 1, bottom).

While previous experiments, which have used a similar setup^23^, have only been able to detect the electric fields from the plasma wave, the authors of the current paper have succeeded in measuring the fields from the electron pulse itself. Analyzing these fields has allowed for a first direct measurement of the size of the electron pulse in the plasma and its evolution at the exit of the plasma—both parameters, which are essential for the further application of these electron pulses or for their injection into a second acceleration stage, either in a conventional accelerator or in a second plasma-based accelerator^24^. In combination with existing diagnostic methods, which are sensitive to plasma-density variations (using optical probe pulses) or to magnetic fields (employing the Faraday effect in the plasma), this novel diagnostic tool, which is additionally sensitive to electric fields both of the plasma wave and of the electron pulse can in the future be used to provide a full characterization of the wakefield and of the electron pulse. By giving us much more detailed insight into the various aspects of the acceleration process this might help to further improve the performance of plasma-based particle accelerators in the future, which would eventually help to bring the envisaged applications of this novel accelerator technology closer to reality.
